# Cutaneous T-cell lymphomas may require an exception to the ABHH consensus regarding empiric vancomycin use in febrile neutropenia

**DOI:** 10.1016/j.htct.2025.103844

**Published:** 2025-05-17

**Authors:** Yung Gonzaga, Jose A. Sanches

**Affiliations:** a National Cancer Institute (INCA), Rio de Janeiro, RJ, Brazil; b Grupo Oncoclínicas, Rio de Janeiro, RJ, Brazil; c University of Sao Paulo Medical School, SP, Brazil

Dear Editor,

We read with great interest the recently published guidelines "Management of febrile neutropenia: consensus of the Brazilian Association of Hematology, Hemotherapy and Cell Therapy - ABHH" by Nucci et al. [1]**.** We fully support the overall recommendations, particularly the more conservative approach to the use of vancomycin as part of the empiric antibiotic regimen, which is well justified by recent epidemiological evidence [2, 3, 4, 5]. However, we would like to highlight a specific subgroup of patients who, in our view, should be considered an exception to the general recommendation against routine empirical anti-MRSA coverage: patients with advanced-stage cutaneous T-cell lymphomas (CTCL), particularly those with Sézary syndrome or extensive mycosis fungoides.

As noted in several studies, these patients have a significantly higher risk of skin and bloodstream infections caused by Staphylococcus aureus, including methicillin-resistant strains (MRSA) with this being one of the main causes of death [6, 7, 8]. The combination of profound immune dysregulation, extensive skin barrier disruption, and frequent colonization with S. aureus places these patients at a distinctively high risk of infections, which may progress rapidly to sepsis and death [9,10]. Additionally, the epidemiological studies cited in the guidelines to support the recommendation against empirical anti-MRSA coverage do not include a sufficient representation of patients with CTCL [2, 3, 4, 5], making it difficult to extrapolate findings for this population.

Given these considerations, we suggest that advanced-stage mycosis fungoides and Sézary syndrome patients should be explicitly recognized as a subgroup that may warrant empirical anti-MRSA coverage in cases of febrile neutropenia until further studies focusing on this specific population bring additional valuable information to optimize their management.

## Author Contribution

**Yung Gonzaga** conceived and wrote the paper. **Jose A. Sanches** wrote the paper.

## Conflicts of interest

Yung Gonzaga received honoraria and speakers' fees from Takeda. Jose A. Sanches received honoraria and speakers' fees from Takeda.
